# A representation-independent electronic charge density database for crystalline materials

**DOI:** 10.1038/s41597-022-01746-z

**Published:** 2022-10-28

**Authors:** Jimmy-Xuan Shen, Jason M. Munro, Matthew K. Horton, Patrick Huck, Shyam Dwaraknath, Kristin A. Persson

**Affiliations:** 1 Department of Materials Science and Engineering, University of California, Berkeley, Berkeley, California 94720 USA; 2 Lawrence Livermore National Laboratory, Livermore, USA; 3 Energy Technologies Area, Lawrence Berkeley National Laboratory, Berkeley, 94720 USA; 4 Energy Sciences Area, Lawrence Berkeley National Laboratory, Berkeley, 94720 USA

**Keywords:** Atomistic models, Structure of solids and liquids

## Abstract

In addition to being the core quantity in density-functional theory, the charge density can be used in many tertiary analyses in materials sciences from bonding to assigning charge to specific atoms. The charge density is data-rich since it contains information about all the electrons in the system. With the increasing prevalence of machine-learning tools in materials sciences, a data-rich object like the charge density can be utilized in a wide range of applications. The database presented here provides a modern and user-friendly interface for a large and continuously updated collection of charge densities as part of the Materials Project. In addition to the charge density data, we provide the theory and code for changing the representation of the charge density which should enable more advanced machine-learning studies for the broader community.

## Abstract (summary)

**Table:** 

Measurement(s)	Electronic charge density
Technology Type(s)	Density-funtional theory

## Background & Summary

The application of Density-Functional Theory (DFT) to many-electron systems has witnessed tremendous growth in the past few decades and has now become the *de facto* simulation tool for physicists, chemists, and materials scientists. The central concept of DFT is that the energy, and in turn all of the physical properties of a quantum system, are completely determined by the electronic charge density of the ground state *ρ*(***r***) with **r** the position vector^1^. The majority of the computational cost in typical DFT calculations is associated with determining *ρ* via an iterative algorithm to arrive at a self-consistent charge density^2^. For the most commonly used exchange-correlation functionals, like the local-density approximation (LDA)^2,3^ and the semi-local functional by Perdew-Burke-Ernzerhof (PBE)^4^, a converged charge density can be used as the starting point for more expensive calculations such as obtaining a detailed bandstructure^5^ or calculating the optical response of the material^6^.

In addition to its central role in standard DFT calculations, the charge density itself is also useful for the analysis of many materials properties. The critical points of the charge density (i.e. where the gradient is zero) are often used as a boundary between atomic neighborhoods. In turn, this allows for a systematic assignment of charge to specific atoms^7,8^, as well as the determination of bonding character between neighboring pairs^9^. Within the realm of energy materials, the charge density can be used as an effective potential to study the migration properties of Li in solid-state materials, as low charge density provides a metric of “free” space in a lattice^10,11^. Consequently, the local minima of the charge density can act as initial guesses for the positions of inserted cations^12^.

A single DFT calculation of the primitive unit cell provides one representation of the charge density within that particular basis set. However, depending on the application, alternative representations might be desired. An important example of this is in machine-learning (ML) algorithms, where obtaining a consistent data representation is essential for deep-learning methods. However, the representation of charge density is heavily influenced by the simulation cell and the Bravais lattice of the periodic structure. Hence, a necessary step in using electronic charge densities in machine-learning applications is to obtain alternative representations of the same charge density that represent the same underlying field. While recent work has examined the effectiveness of representations in Fourier space^13^, any ML investigation of local interaction (e.g. adsorption and intercalation of ions) requires flexible representations in real space. Towards that end, our framework will provide code to obtain arbitrary real space representations of charge density for a given material directly from a DFT-computed charge density.

The charge density of any crystalline solid, and indeed any periodic field, is naturally represented in a plane-wave basis set, where the inherent periodicity of the system is embedded in the underlying representation. For a sufficiently converged finite plane-wave basis, the continuous charge density *ρ*(**r**) and its Fourier transform *ϕ*(**k**) can be accurately sampled by a three-dimensional array indexed by *i*, *j*, and *k* with *N*_1_, *N*_2_, and *N*_3_ evenly spaced grid-points along each lattice vector, and can be converted from one to the other via a discrete Fourier transform

1 $$\rho \left({\bf{r}}\right)\equiv \left({{\bf{a}}}_{1},\;{{\bf{a}}}_{2},\;{{\bf{a}}}_{3},\;{\rho }_{i,j,k}\right)\ \underset{{{\mathcal{F}}}^{-1}}{\overset{{\mathcal{F}}}{\iff }}\ \left({{\bf{b}}}_{1},\;{{\bf{b}}}_{2},\;{{\bf{b}}}_{3},\;{\phi }_{i,j,k}\right)\equiv \phi \left({\bf{k}}\right).$$

where **a**_*α*_ and **b**_α_ represent the real and reciprocal lattice vectors, and *i*, *j*, and *k* are the indices of regularly-spaced grid points along the lattice vectors. Due to the discrete nature of numerical Fourier transforms, the number of grid points of a real-space representation is always equal to the number of plane waves needed to represent the data in reciprocal space.

A representation of the charge density is uniquely determined by three vectors and a scalar matrix either in real or reciprocal space. Each representation only provides a “view” of the infinite periodic data represented in a specific unit cell and an infinite number of such representations exist for a given charge density. Regardless of the grid size and the periodic cell representation, the DFT-computed charge density represents the same underlying field, yet it is routinely recomputed when any change is needed in the representation, even when the computational parameters are unchanged, often at considerable expense. One common example is the use of the electrostatic potential of a super cell to correct for the periodic image effects of a charged defect^14^.

Due to the significant amount of computational resources devoted to computing the electronic charge densities, as well as the growing domains of their application, especially for the training of data-intensive machine learning models, there is a pressing need for a large-scale representation-independent database of charge densities. The Materials Project (https://materialsproject.org) - as a rapidly growing (currently more than 215,000 users) materials informatics resource - is a natural platform for the dissemination of such data. The structural and thermodynamics information of solid-state materials are available across other quantum chemical databases such as AFLOW^15^, NOMAD^16^, and OQMD^15^, and a targeted charge density dataset of materials with cubic symmetry has recently been published^17^. Our work aims to be the first set of publicly available charge density data for periodic systems without constraints on the structural family. The data and API will be maintained as part of the Materials Project ecosystem. The work presented in this article provides details on how the charge densities in our database are computed and how they can be accessed. In addition, we provide a high-level API for querying and post-processing the charge density data. Among other features, the API will allow users to take an existing atomic structure and query for charge density of the same material, in the representation/view of the user’s choosing.

## Methods

In this section, we provide details on the scope of the charge densities database and the precise set of computational parameters used to generate the data. Additionally, we will demonstrate features of the API that allow users to obtain arbitrary views of the charge density data, including up-sampling/compressing the data via Fourier analysis and symmetry operations like translations, rotations, and super cell transformations.

### Calculation parameters

The charge densities are obtained from DFT calculations performed using the static calculation workflow within the atomate software package^18^, and relaxed input structures from the Materials Project (MP) database^19^. The projector-augmented wave (PAW) method as implemented in the plane-wave Vienna Ab-initio Simulation Package (VASP) is used in conjunction with the PBE generalized-gradient approximation functional^4^. The default set of MP calculation input parameters was used, which have been demonstrated to produce well-converged results^20^. Included in these parameters is an energy cutoff of 520 eV, a total energy error threshold of 5 × 10^−5^ eV/atom, and a reciprocal *k*-point density of 100/A^−3^. The only addition made to the input set is to enable aspherical contributions in the gradient corrections inside the PAW spheres. Hubbard *U*-corrections are included with materials containing oxygen and fluorine. Elements Co, Cr, Fe, Mn, Mo, Ni, V, and W use values of 3.32, 3.70, 5.30, 3.90, 4.38, 6.20, 3.25, and 6.20 eV, respectively.

### Changing the charge density representations

**Fig. 1:**
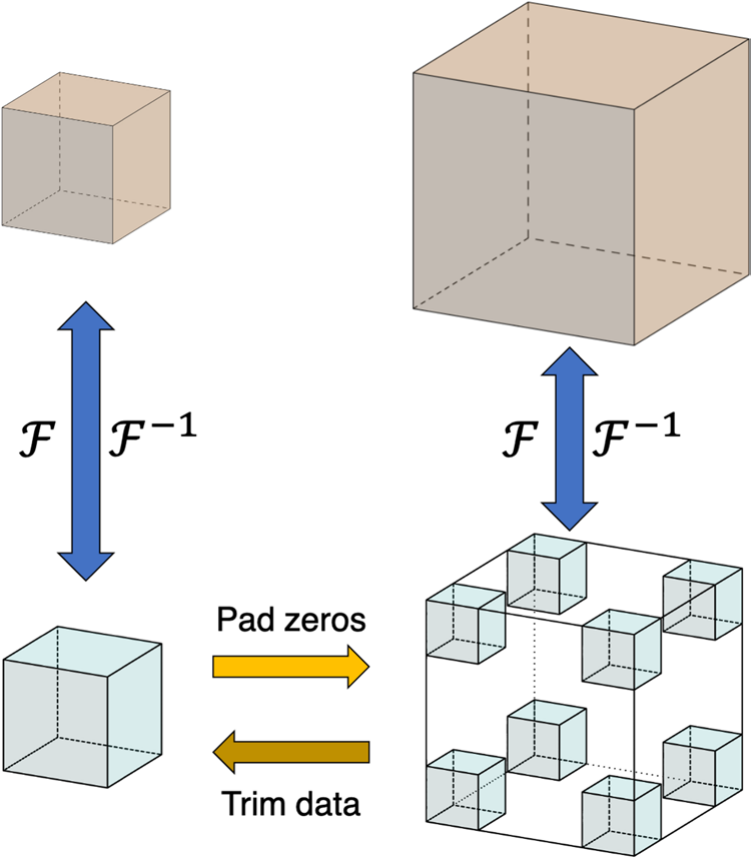
Schematic of data transfer for Fourier interpolation and compression. The more densely sampled (larger) and the coarsely sampled (smaller) 3D blocks of real-space data can each be transformed to Fourier space, resulting in a Fourier representation of the same size. To up-sample the data, we use the smaller block in Fourier space, augment with zeros while keeping all the data fixed near the origin (at the corners of the cube). To compress, we crop the data in Fourier space and perform an inverse Fourier transform.

Given one representation $({{\bf{a}}}_{1},{{\bf{a}}}_{2},{{\bf{a}}}_{3},{\rho }_{i,j,k})$ of the charge density *ρ*, we may transform it to any other representation $({{\bf{a}}}_{1}^{{\prime} },{{\bf{a}}}_{2}^{{\prime} },{{\bf{a}}}_{3}^{{\prime} },{\rho }_{{i}^{{\prime} },{j}^{{\prime} },{k}^{{\prime} }})$ by resampling the data. Due to computation time and data storage constraints, DFT codes will typically use the fewest grid points possible to represent the charge density which limits the effectiveness of local interpolation schemes. However, since our charge densities have periodic boundary conditions and are reasonably smooth (owing to the use of pseudo-potentials), the charge density can be first represented in Fourier space and then interpolated. We can up-sample our data via Fourier interpolations^21^ as shown in Fig. 1. The procedure to perform Fourier interpolation of real space data is as follows:

1. Take the discrete Fourier transform of *ρ*_*i,j,k*_.
2. Augment the resulting Fourier data *ϕ*_*i,j,k*_ with zero-valued higher frequency components.
3. Apply the reverse transformation to obtain the up-sampled data.

The augmented Fourier data is mathematically equivalent to the original Fourier data. Thus, the inverse transform of the augmented Fourier data must be equivalent to the original real space data sampled at a higher density. Increasing the grid density using Fourier interpolation enables us to up-sample *ρ*_*i,j,k*_ in each direction by a factor of *γ*_u*p*_. We may then resample the local grid with a linear interpolation scheme to ensure the fidelity of our data.

Given a primitive-cell representation of the charge density — $({{\bf{a}}}_{1},{{\bf{a}}}_{2},{{\bf{a}}}_{3},{\rho }_{i,j,k})$, any periodic representation of a scalar field *f*(**r**) can be understood as applying an arbitrary translation on the unit cell by a vector **t**:

2 $${\widehat{T}}_{{\bf{t}}}\,f({\bf{r}})\equiv f({\bf{r}}-{\bf{t}}),$$

followed by a super cell transformation $\widehat{P}$ defined as an integer matrix with ${\rm{\det }}(\widehat{P})\ge 1$ which acts on the lattice vectors from the right

3 $$({{\bf{a}}}_{1}^{{\prime} }\;{{\bf{a}}}_{2}^{{\prime} }\;{{\bf{a}}}_{3}^{{\prime} })=({{\bf{a}}}_{1}\;{{\bf{a}}}_{2}\;{{\bf{a}}}_{3})\widehat{P}.$$

**Fig. 2:**
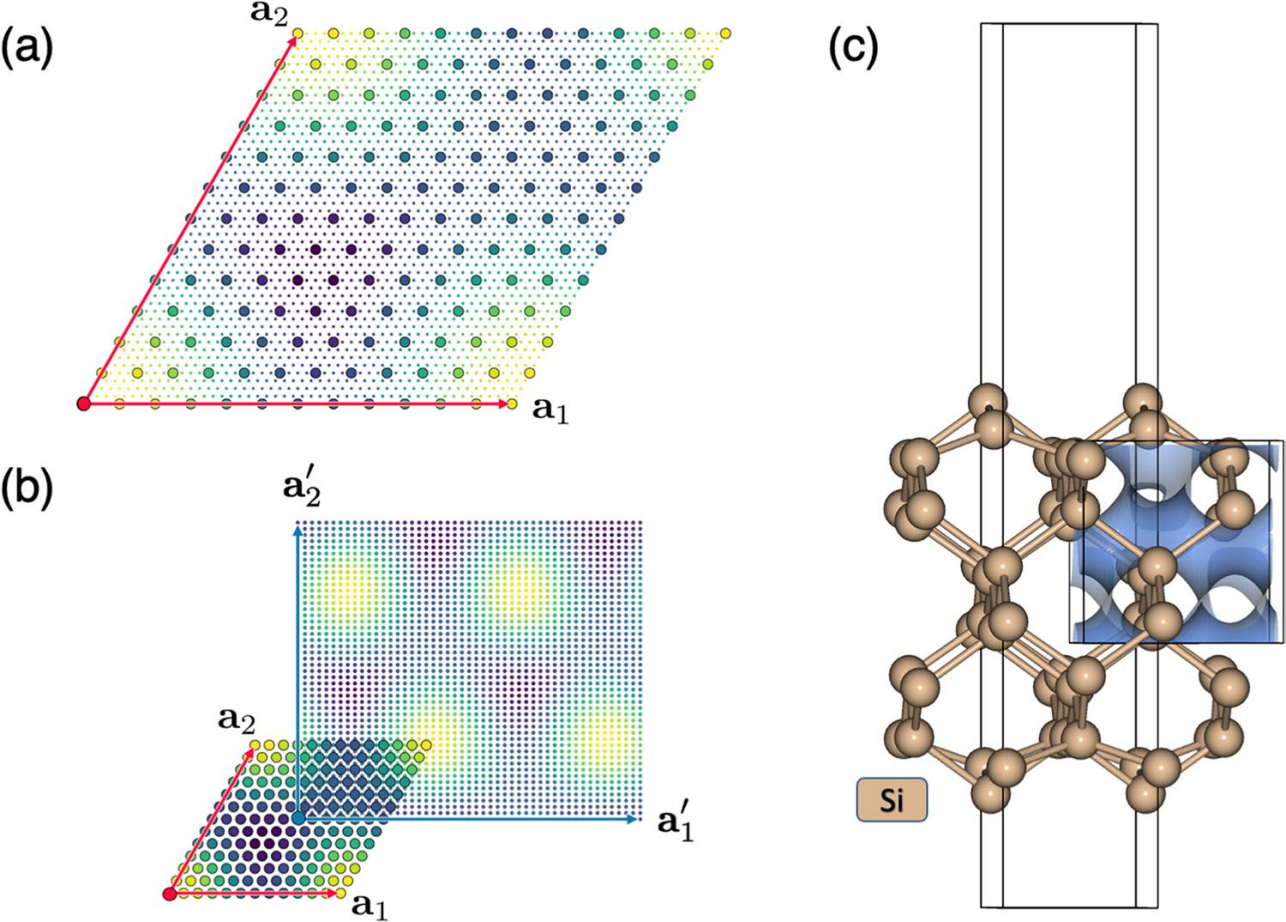
Periodic re-gridding applied to a 2D slice of the charge density for a two atom Si unit cell where each large circle corresponds to a data point in the original 12 × 12 grid. The result of Fourier-interpolating the data in the original unit cell onto a 48 × 48 grid is shown in (**a**). The transformed periodic representation ${{\bf{a}}}_{1}^{{\rm{{\prime} }}}=2{{\bf{a}}}_{1}$ and ${{\bf{a}}}_{2}^{{\rm{{\prime} }}}=2{{\bf{a}}}_{2}-{{\bf{a}}}_{1}$ with a shift of ${\bf{t}}=({{\bf{a}}}_{1}+{{\bf{a}}}_{2})/2$ and a grid size of 48 × 48 is shown in (**b**). A cubic crop of a Si slab calculation is shown in (**c**).

Our software is capable of performing the same operations in arbitrary dimensions. As an example, in Fig. 2, we show the results of re-griding using a 2D slice of the charge density in a two-atom Si unit cell which only cuts across a single atom at the origin, Fig. 2(a) shows the result of Fourier interpolating the field from a 12 × 12 grid (large circles) onto a 48 × 48 grid (smaller circles). In Fig. 2(b), the modified representation is obtained by first shifting the origin to the center of the cell at **t** = (**a**_1_ + **a**_2_)/2 followed by a change of basis to ${{\bf{a}}}_{1}^{{\prime} }=2{{\bf{a}}}_{1}$ and ${{\bf{a}}}_{2}^{{\prime} }=2{{\bf{a}}}_{2}-{{\bf{a}}}_{1}$.

While integer-valued super cell transformations will yield an equivalent periodic underlying charge density, non-integer basis transformations are used to obtain an arbitrary crop of periodic charge density sampled at any density. As an example, we show how a non-periodic cubic sample of the surface charge density can be obtained from the slab calculation in Fig. 2(c). The simulation was performed using a 7.73 Å  × 3.87 Å  × 21.88 Å orthorhombic Si slab cell and the charge density is stored on a 120 × 60 × 336 grid. A 5 Å  × 5 Å  × 5 Å cropped region of the charge density sampled on a 48 × 48 × 48 grid is indicated by the blue iso-surface in Fig. 2(c). It is important to note that the cropped cell can exceed the boundaries of the original simulation cell in any direction. In the example, the smallest dimension of the simulation cell is 3.87 Å while the cropped cube has side lengths of 5 Å. This feature essentially allows us to obtain the charge density in any preferred real-space dimensions, independent of the simulation cell parameters. Additionally, this allows us to freely choose the simulation cell in situations where periodic-image effects are not important.

### Database details

**Fig. 3:**
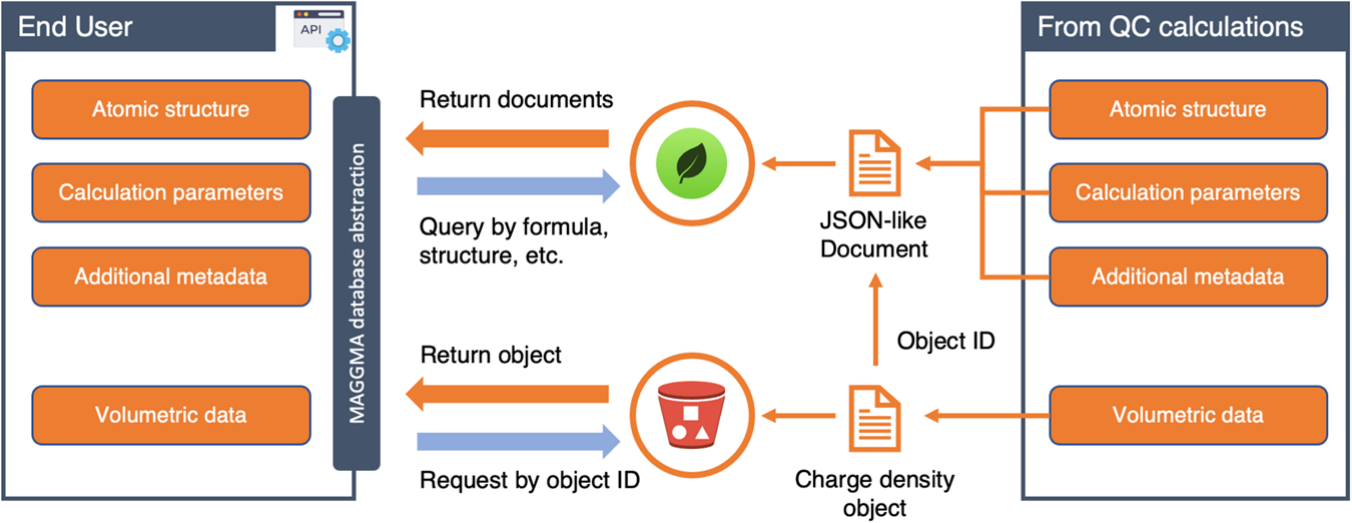
Data pipeline for the charge density database, illustrating how the output files from quantum chemistry calculations are stored and accessed. Basic data are first converted into a JSON-like format to be stored on a MongoDB server, which allows queries on any of the stored fields. The charge density data is converted into an array-like object with additional meta-data (e.g. ObjectID) and stored in an AWS S3 bucket. Since the ObjectID is stored as a field in the MongoDB database, the API is able to combine the MongoDB data with the according S3 object and reconstruct the original data.

We use a hybrid data model to serve the data: Queryable data such as chemical formula, total energy, and calculation parameters are served as JSON-like documents using MongoDB, while much larger and not-queryable charge density data is served using AWS S3 object storage^22^. When a charge density is parsed from the output file to a serialized object, a unique Object ID is assigned and stored alongside the other data in the MongoDB database. From the client’s perspective, two concurrent requests are made: one to obtain calculation inputs and outputs from MongoDB, and another for the charge density data from the S3 bucket. A visual representation of the data flow is provided in Fig. 3.

## Data Records

The dataset itself can be viewed through the Materials Project website www.materialsproject.org^23^. The raw charge density data output from DFT calculations can be obtained from the corresponding MP API endpoint: https://api.materialsproject.org/charge_density. Each entry can be referenced with a particular DOI through the associated MP material entry. Additionally, the input parameters for the specific calculation used to generate the entry can be obtained from the tasks endpoint at https://api.materialsproject.org/tasks. Details for how to interact with the referenced endpoints can be found in the [sec:usage]Usage Notes section.

## Technical Validation

We can elucidate the performance of the re-griding algorithm using a larger set of elemental polymorphs from the Materials Project. For this test set *S*_e*l*_, we selected 117 single-element structures from MP for which the energy above the convex hull was less than 1 *μ* eV and the number of atoms in the unit-cell was less than 20. For each structure in *S*_e*l*_, we perform VASP static calculations on the primitive unit cell and on two super cells created by:

4 $${\widehat{P}}_{1}=\left(\begin{array}{lll}1 & 1 & 0\\ 1 & -1 & 0\\ 0 & 0 & 1\end{array}\right),$$

and

5 $${\widehat{P}}_{2}=\left(\begin{array}{lll}2 & 0 & 0\\ 0 & 2 & 0\\ 0 & 0 & 2\end{array}\right).$$

**Fig. 4:**
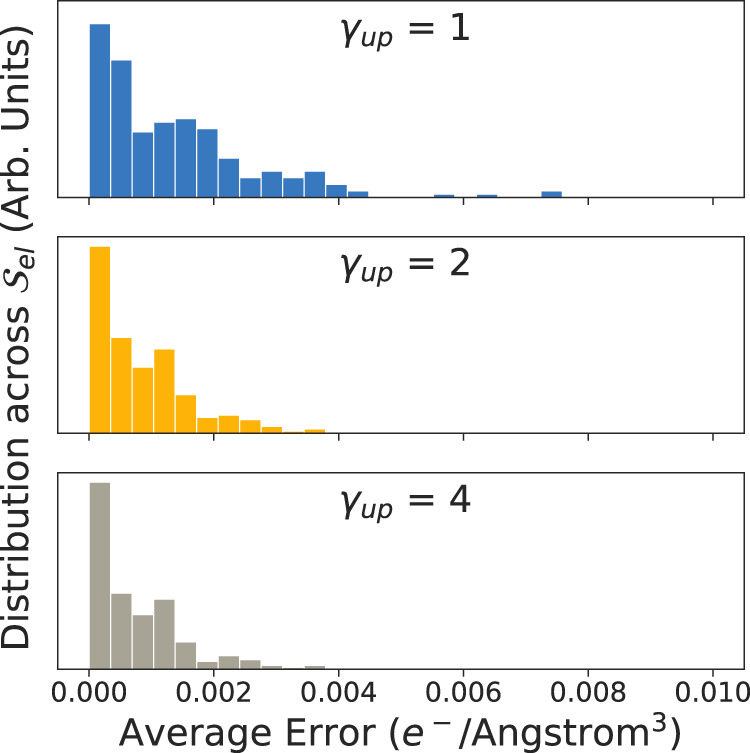
Distributions of the errors between re-sampled charge density and explicitly calculated charge densities.

For each charge density obtained using an explicit super cell calculation, we obtain the average error compared to a super cell charge density obtained from transforming the unit cell charge density. The results of the comparison are shown in Fig. 4. We observe that using an up-sampling factor of *γ*_u*p*_ = 4, the transfored and explicitly calculated pseudo-charge density, whose values can ranges from 0 in vacuum to 100 e^−^/Å^3^ near the atomic cores, only exhibits a difference of <0.002 e^−^/Å^3^. The data and code used in the validation procedure can be accessed as a direct download^24^.

## Usage Notes

**Fig. 5:**
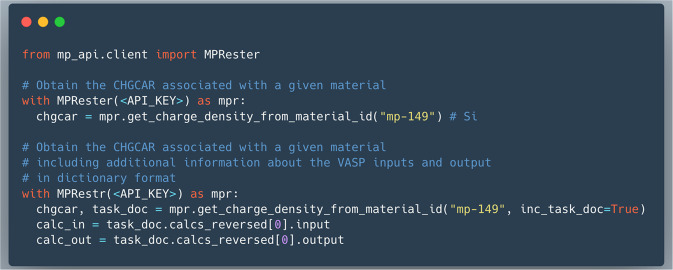
Example code for querying the charge density database.

To facilitate access to this data, convenience functions have been implemented as part of the Materials Project REST API client. These are contained within the MPRester class as part of the pymatgen software package^19^. More specifically, the member function get_charge_density_from_material_id is provided to send requests to the API endpoints. This function takes as input the Materials Project ID associated with a given material in the database and returns a Chgcar object. If the inc_task_doc flag is set to True, an additional task document containing all of the calculation details will also be returned. The VASP calculation that produced the charge density is always the last calculation that corresponds to the zeroth entry in the calcs_reversed list. And the inputs and outputs are stored as dictionary key-value pairs under input and output attributes. With the MPRester class imported, the code workflow in Fig. 5 can be used to query for the charge density and calculation details.

In order to alter the representation of the charge density obtained from the API endpoint, the pyRho python package https://github.com/materialsproject/pyrho can be used alongside the pymatgen Chgcar object obtained from the API query. Examples of how to re-grid, interpolate, and visualize are included in the repository as a set of Jupyter^25^ notebooks.

## Data Availability

Access to the charge density data provided by the Materials Project API (https://github.com/materialsproject/api) and grid transforms of the charge density is done using the pyRho python package. See the [sec:usage]Usage Notes section for more information. The scripts used to generate the validation data can be access at along with the direct download of the validation dataset^24^
